# Intelligent Prediction and Rural Financial Development Based on Abnormal Detection of Sensor Data

**DOI:** 10.1155/2022/6404825

**Published:** 2022-02-28

**Authors:** Jiong Liu

**Affiliations:** Department of Tourism and Commerce, Xuancheng Vocational and Technical College, Xuancheng, Anhui 242000, China

## Abstract

Wireless sensor network is a multisensor wireless network system, which consists of multiple sensors and is configured independently. Because the network generates a large amount of data, the frequency, performance, and computing power of sensor nodes are limited, and they are particularly vulnerable to harsh environments and malicious attackers. This leads to the occurrence of malicious nodes, emergencies, and abnormal data in the sensor network system. Failures can also have a significant impact on sensor network services. The two main functions of wireless sensor network security are abnormal node detection and data anomaly detection. These two directions are mutually independent and complementary. Therefore, under the promotion of the rural revitalization strategy and the precision poverty alleviation strategy, China has increased its agricultural efforts. At this stage, all localities focus on the construction of rural financial systems to ensure that scattered farmers and rural small and micro-enterprises receive comprehensive financial services. The establishment of a rural financial system based on “intelligent forecasting” can improve financial development theories and build new ideas for rural financial development. And, the balance between realization and profitability, and then, through the use of Internet technology to make traditional financial institutions more effective in providing financial services, new online financial platforms can use them to make up for the existing shortcomings of traditional financial institutions as much as possible. In this article, through the research on the intelligent prediction of sensor data anomaly detection, it is applied to the development of rural finance and promotes the development of rural finance.

## 1. Introduction

In recent years, with the rapid development of computer technology, electronic equipment, wireless networks, and communication systems, wireless sensor networks have continued to emerge and have gradually become an important channel for people to obtain data and information [1]. Wireless sensor networks have the characteristics of limited node sources and are easy to break [2]. Although technologies such as mandatory passwords and secure routing have improved the security of sensor networks, there is still a lack of effective methods to detect abnormal information in the sensor network, making the sensor network more effective. Protection becomes difficult [3]. The improvement of data anomaly detection technology can greatly promote the future application and development of sensor networks. Each sensor node maintains the trust value of neighboring nodes to reflect its previous decision-making behavior [4]. Two thresholds are used to reduce the false alarm rate and increase the incident. Area detection accuracy is used to achieve more accurate detection of malicious nodes without sacrificing normal nodes [5, 6]. The simulation results show that the two-threshold scheme is better than the single-threshold scheme [7]. After screening and removing malicious nodes in the sensor network, a fault detection method based on a distributed multilayer wireless sensor network is introduced [8]. Through the analysis of false datasets and real-time environmental data collection data, it is shown that compared with the central system and the reference system, the improved anomaly detection program can achieve a higher detection rate, false alarm rate, and lower communication utilization [9]. Finance conducted a “Internet + Intelligent Forecasting” research. Using the concepts and theories related to the overview, firstly, it analyzes the status quo of rural financial development under the background of “Internet + intelligent forecasting” from the service functions of traditional rural financial institutions and the service functions of Internet rural financial institutions; Alibaba and CreditEase analyze and demonstrate the three typical rural finance “Internet + smart forecasting” cases to obtain development information: clarify the “Internet + smart forecasting” agricultural development strategic goals, make full use of big data, and promote the self-improvement of local financial institutions; By further analyzing the problems existing in the development of rural finance in the context of the “Internet,” we found that there are various problems in rural financial risks. It is necessary to further expand the rural credit control system, improve Internet finance laws and regulations and rural network infrastructure, and increase the penetration rate of farmers' financial knowledge [10]. Finally, it puts forward targeted countermeasures to promote the development of rural finance under the background of “Internet + intelligent forecasting,” establishes a political incentive mechanism for state intervention, strengthens the prevention and monitoring of rural financial risks, and promotes financial publicity and education in rural areas [11, 12].

## 2. Related Works

The literature introduces the application background of wireless sensor networks, describes the research background and practical significance of malicious nodes and abnormal data detection, analyzes the current status of the investigation of malicious messages and abnormal information detection technologies at home and abroad, and provides a complete, structured, and summarized summary [13]. The literature introduced the basic problems of wireless sensor networks, introduced the anomaly detection datasets commonly used in wireless sensor networks, and explained the architecture of wireless sensor networks in detail. The literature introduces a two-step detection system for malicious nodes on wireless sensor networks. Each sensor node maintains the trust of neighboring nodes to reflect its past behavior in the decision-making process [14]. The simulation results show that the two-tier plan is better than the single-tier project. The program provides a set of parameters whose values can be adjusted according to the application system to maximize efficiency. The literature introduces an abnormal data detection method for distributed wireless sensor networks based on hierarchical aggregation [15]. Through simulation analysis on artificially constructed datasets and datasets collected in real environments, it shows improved anomaly detection schemes and centralized schemes. Compared with the HSCBD scheme as a control, it can achieve a high detection rate and a low false alarm rate and reduce communication consumption. The literature introduces a distributed error detection system [16]. The algorithm includes two main steps: self-detection and intermediate pod detection. The algorithm makes full use of the spatiotemporal data connection of data distribution in wireless sensor networks and divides abnormal data into two steps: time and location [17]. Carry out anomaly detection process on the basis of reliability improvement.

## 3. Design and Implementation of the Intelligent Prediction Method Based on Abnormal Detection of Sensor Data

### 3.1. Analysis of Wireless Sensor Network Architecture and System Requirements

#### 3.1.1. Wireless Sensor Network Architecture

A sensor is usually a cheap device with relatively low battery life, computing power, and storage capacity. The main purpose of the network is to identify, store, process, and transmit environmental information in the area covered by the system. Related technologies mainly include three major technologies: sensor technology, information technology, and communication technology. The three architectural components of a wireless sensor network are sensors, sensing objects, and users.

**Figure 1:**
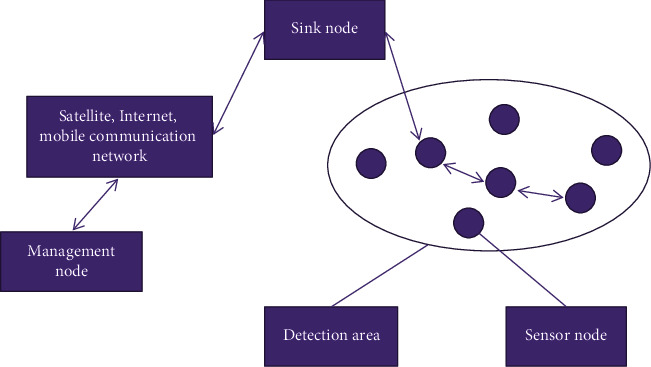
Typical wireless sensor network architecture diagram.

There are many types of wireless sensor nodes, but a single node has a single function. It can generally be used to measure various indicators in the natural environment, such as earthquake, noise, temperature, humidity, and light intensity. It has a wide range of application prospects and is used in various parts of today's society.  Figure 1 is a typical wireless sensor network architecture diagram, in which the sensor nerves are automatically distributed in the area of interest manually, and they work together to collect and process the data in the coverage area and transmit all or part of the data to the sewer or base station. After the summary data are sent, the measurement data can be processed into multiple items. The base station sends the metadata collected through satellite or wireless network back to the administrator for decision-making. The administrator can also send instructions to the base station through the network and send the instructions to each relevant sensor.

Most sensor nodes are very small. Because the technology development is not very mature, the computing power, memory, and communication capabilities of the nodes are limited. Network sensor systems are often used in unintentional or difficult environments, and the battery cannot be replaced, and the life cycle is relatively short. Many wireless sensor network power supply modules include wireless communication modules, processing modules, and sensors. With the development of single-chip technology and electronic integration technology, computer power consumption has been greatly reduced. In addition to collecting and processing local data, sensors must also cooperate with other entities to perform certain tasks under special conditions.

The convergence node is an intermediary between the sensor network and the external Internet, and can identify the exchange protocol between the Internet of Things and the Internet, processed by the network sensor, and sent to the management node after collection and processing.

The management node is the “stand-in” of the entire sensor network and generally directly controls the sensor identification system of the sensor network through it, and the network administrator can access the review intelligence through management, decision-making, and guidance clauses.

#### 3.1.2. The Internal Node Composition of the Wireless Sensor

The wireless sensor network consists of a large number of randomly distributed sensors. For example, if we conduct an environmental audit, the data will collect various information in the environment (such as temperature, humidity, and air pressure) to form a self-hosted wireless network. Each sensor is sent finally to the management terminal and configuration terminal. There are two types of nodes in the network: ordinary sensor nodes and gateway nodes. Distributed sensor networks can communicate with each other and connect to a gateway node through multiple data. The gateway node collects and transmits data. The network can access the Internet through the gateway node. Communication with the entire site of the data controller and the entire system can be managed and controlled from the headquarters.

**Figure 2:**
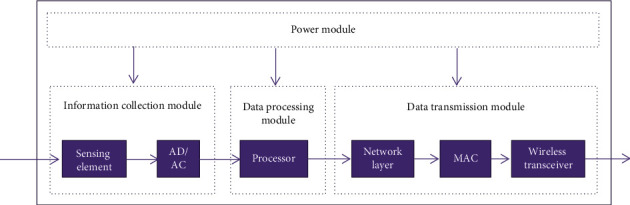
Structure diagram of the internal composition of sensor nodes.

The internal structure of the sensor is shown in Figure 2. The data acquisition module is composed of two subunits: the sensor component and the analog-to-digital converter (AD/DC). The digital output signal is transmitted to the data processing module through the analog-to-digital converter. The power supply module supplies power to each sensor module and usually uses a battery to power the device. When designing sensor hardware components, save device power as much as possible and extend the life of the device. It is necessary to use components with the lowest power consumption possible, and the sensor should not communicate. Turn off some communication functions when needed. When developing communication software for sensor networks, you must consider the energy consumption of the network. By eliminating some unimportant network performance indicators, you can achieve higher power utilization and extend the life of the entire network.

#### 3.1.3. Data Security Requirements of Sensor Networks

Wireless sensor networks have different security requirements in different application scenarios. Aiming at sensor network systems commonly used in industrial and agricultural production, this article divides the most important security requirements of wireless sensor networks into basic security requirements and network system security requirements (see Table 1).

The threats to wireless sensor networks are caused by environmental factors such as natural disasters and intruders, and face different threats in different fields such as data, communications, services, and hardware, as shown in Table 2.

Wireless sensor network nodes are large in number and widely distributed. In practice, sensor networks are usually deployed in areas where few or no people are on duty. In addition, the hardware fragility of sensors and some potential software defects cause further damage to the network.

An information attack is an attacker maliciously intercepting, threatening, accessing, or destroying data in the network, resulting in incorrect judgments of network data or errors in data reporting. False information is confusing and difficult to be discovered by people. Therefore, such attacks are very hidden and dangerous and should be considered. In response to such attacks, the sensor network data detection technology cannot analyze the dataset by the wireless sensor network to determine whether the node data are abnormal, thereby reducing the harm of data attacks.

Attackers perform Sybil attacks by invading network nodes and then using authentication technology to impersonate multiple legitimate network nodes. This attack will destroy the network topology and disrupt normal network communication. Vulnerability attacks are mainly related to the attacker manipulating data to be sent after the intrusion part or all of the data are related, resulting in the loss of a large amount of normal data on the network and abnormal communication between messages. Most flood attacks have two attack methods. One attacker controls the node to continue trying to establish a connection with the attacked node to use the attacker's node connection resources, and the other is that the attacker controls the node to continuously send invalid data to the attacked node. The request is forwarded by the attacked node, thereby destroying the computer and frequency band node resources. Both of these attack methods prevent the attacker from processing legitimate requests from other data and greatly increase the energy consumption of the attacked node. Most wormhole attacks target two malicious nodes that together form a communication tunnel. Even if the two malicious nodes are far apart, there is only one step between the two malicious nodes. In this way, the hop times of the two nodes are shorter than normal, so as to achieve the purpose of deceiving the right of way and destroying the normal routing path.

Service attacks are mainly carried out through distributed denial of service (DDOS) attacks. The purpose is to exhaust the communication resources or computing resources of the sensor network, causing the system service group network to be unavailable.

The purpose of hardware attacks is to control smart devices in a sensor network by destroying device hardware or intruding devices. Direct damage to such devices will cause some system functions to be paralyzed. Smart device monitoring can be used as a spring trigger to attack sensor network systems, such as managing sensor data through data transmission, sending false monitoring data to attack network readiness, running malicious code to attack other devices, and preventing hardware attacks by installing protective shells and other security measures. It can prevent the physical security of network equipment. Using fault detection technology, a strict access control system can be established to detect faulty equipment in time and prevent intruders from entering the equipment.

Anomaly attributes are various network status indicators that appear when the sensor network is threatened. In order to use the K method to detect anomalies, information objects need to be grouped according to the anomaly attributes. Therefore, the basic principle and description of character selection is to confirm the K-mean algorithm detection result important factor.

Aiming at the threat of data attack, through analyzing typical data attack methods, the selected characteristics are shown in Table 3.

The data collected by sensor nodes generally have several dimensions. Through the analysis of multiformat databases, the spatial interaction of sensor network data can be effectively used to detect data errors. The data can reflect the feasibility and statistical characteristics of sensor data flow. By introducing the work of this group of analysis, the statistical (time-related) characteristics of the dataset can be used to detect anomalies and improve the discovery results of the search system.

### 3.2. Research on Anomaly Detection Algorithm of Sensor Data

#### 3.2.1. Standardization of Sensor Data

A sensor node can be composed of multiple sensor elements, and each discovery element is responsible for collecting one or more types of data. Therefore, the data center can store various types and different scales of information, such as temperature, humidity, air pressure, and brightness. This information has its own format and data count. If you cannot combine different data formats and dimension units, you cannot use multidimensional data horizontally. Therefore, if you want to use multiple datasets to perform sensor data anomalies, you need to normalize the data in various formats and then calculate the Euclidean distance between different data objects, which is used to measure the similarity of the data. The following anomaly detection methods in this article require standard data object management. A variety of data normalization methods have been proposed at home and abroad, among which the most commonly used in research are “minimum-maximum normalization,” “Z-score normalization,” “normalize by decimal calibration” and so on.

Then, calculate the minimum-maximum normalization by the following formula:

(1) $$\begin{array}{c} x^{\prime }=\frac{x-\underset{X}{\min}}{\underset{x}{\max}-\underset{x}{\min}}\left(\overset{'}{\underset{X}{\max}}-\overset{'}{\underset{X}{\min}}\right)+\overset{'}{\underset{x}{\min}}. \end{array}$$

Z-score uses the standard method and the deviation of the original dataset to standardize the data. When the maximum and minimum values of a certain attribute in the dataset are unknown or unpredictable, this method can be used for regular data.

The standardized formula for calculating Z-score is shown in the following formula:

(2) $$\begin{array}{c} X^{\prime }=\frac{X-\bar{X}}{\sigma_{J}}. \end{array}$$

The regularity of decimals is standardized by moving the decimal point of the *X* attribute. The absolute maximum value of the *X* attribute determines the number of decimal places that can be moved. Assuming that the value of the original data *x* of some *X* attributes in the dataset is assumed to be *x*′, the following formula indicates

(3) $$\begin{array}{c} x^{\prime }=\frac{x}{10^{j}}. \end{array}$$

Because the range of data collected by wireless sensors is relatively fixed, the maximum and minimum values of data can be predicted based on objective physical facts, and a significant maximum value can be set for each attribute in advance, and the minimum value of the linear transformation of the original data can be selected—maximum normalization method calibration data.

Shannon used statistical probability methods to give the definition of information entropy:

(4) $$\begin{array}{c} H\left(X\right)=-\sum_{i=1}^{n}p\left(x_{i}\right)\log\left(p\left(x_{i}\right)\right). \end{array}$$

The data collected by the sensor node T changes continuously with the change in the data retention time. Generally, the time t data are related to historical and subsequent data. The selection of the data collection period has a greater impact on this relationship. For a single sensor stream, the estimated time of collection is T, and the duration of the sensor flow can be expressed by the following formula:

(5) $$\begin{array}{c} X\left(t\right)=\left[\ldots ,x\left(t-\Delta T\right),x\left(t\right),x\left(t+\Delta T\right),\ldots \right]. \end{array}$$

The sliding window model uses a sliding window of length W (*w* > 0) to divide the sensor data stream into window data and data outside the window, and the window contains W sample data. When the window slides, the data before the previous sampling time t exit the window, and the information of the next sampling time will appear in the window. Assuming that W and W are two adjacent windows, and the window scrolling distance is 1, the dataset (t) before its movement can be expressed by the following formula:

(6) $$\begin{array}{c} X_{1}\left(t\right)=\left[x\left(t-W^{*}\Delta T\right),\ldots ,x\left(t-\Delta T\right),x\left(t\right)\right]. \end{array}$$

The data sequence *X*(*t*) after moving the sliding window can be expressed by

(7) $$\begin{array}{c} X_{2}\left(t\right)=\left[x\left(t-\left(W-1\right)*\Delta T\right),\ldots ,x\left(t\right),x\left(t+\Delta T\right)\right]. \end{array}$$

The data spacing (distance) is determined by the Euclidean distance, which is used to represent the actual distance between two data objects in the n-dimensional space. It can be used as a measure of the similarity of data objects. Generally speaking, the smaller the distance, the greater the similarity. For data objects *X* (*t*) and *X* (*t*), the distance can be expressed by the following formula:

(8) $$\begin{array}{c} D\left(X_{1}\left(t\right),X_{2}\left(t\right)\right)=\sqrt{\overset{n}{\underset{k=1}{\sum }}{\left(x_{1k}-x_{2k}\right)}^{2}}. \end{array}$$

For the information entropy *h*(*t*) and *h*_2_(*t*) of the data sequence, the distance can be expressed by the following formula:

(9) $$\begin{array}{c} D\left(h_{1}\left(t\right),h_{2}\left(t\right)\right)=\left|h_{1}\left(t\right)-h_{2}\left(t\right)\right|. \end{array}$$

Information entropy sequence calculation is given as follows:

(10) $$\begin{array}{c} p_{i}=P\left\{X=x_{i}\right\}=\frac{\text{count}\left(x_{i}\right)}{\sum_{i\geq 1}\text{count}\left(x_{i}\right)}. \end{array}$$

Calculate the entropy of the sliding window information as a function of sampling probability:

(11) $$\begin{array}{c} h_{j}=\sum_{i21}p_{i}\text{log}\frac{1}{p_{i}}. \end{array}$$

While changing the window, the information of the window is calculated sequentially, so the duration of the information can be expressed by the following formula:

(12) $$\begin{array}{c} H\left(t\right)=\left\{h_{1},h_{2},h_{3},\ldots ,h_{j},\ldots \right\}. \end{array}$$

#### 3.2.2. Data Anomaly Detection Indicators

There are two main sources of anomalies in wireless sensor networks, namely, faults and events. The fault is the reading when the noise measurement value or the sensor fails. This kind of abnormality often occurs. The possibility of abnormality caused by the actual event is usually very low. Bad data usually show that the change in some data is significantly different from other data. Since bad data will affect the quality of the dataset, it should be detected and corrected as much as possible. Such anomalies usually last for a long time and change the normal trend of sensor data. However, if the sensor fails, a similar persistent anomaly will also occur, which makes it difficult to distinguish these two different types of anomalies only by looking at the sensor data stream from the node itself. In this case, anomaly detection technology may need to use the spatial similarity of sensor data, such as data from neighboring nodes, because, in general, sensor failures are not spatially related, while events are the opposite.

Supervised and semisupervised methods require a set of preclassified normal and abnormal data, and all normal and abnormal feature information is obtained in the learning stage, and then, the test data are compared with this learned classification prediction model. However, this kind of preclassified data is not always available, and it is difficult to obtain in real wireless sensor network applications. Although there is a preclassifier that can handle historical data well, it may not be able to distinguish between new normal data and abnormal data. In contrast, unsupervised methods do not require prelabeled data. On the contrary, some statistical data are used to identify abnormal. For example, in the nonremote control method, the normal mode is determined by the average distance between each data and its neighbors. If the distance between the measured values of the data provided to the nearest k-th neighbor is greater than this number, the data are considered anomaly, compared with supervised or semisupervised methods, unsupervised methods are more suitable for wireless sensor networks.

Table 4 shows the possible situations of abnormal data detection results of the wireless sensor network.

Wireless sensor networks usually use the following indicators to measure the effect of abnormal data detection: DR, FPR, FNR, and the quantitative indicators can be defined as follows.

DR is defined as the rate at which abnormal data are correctly detected as abnormal, expressed as

(13) $$\begin{array}{c} \text{DR}=\frac{\left\|\text{TN}\right\|}{\left\|\text{FP}\right\|+\left\|\text{TN}\right\|}. \end{array}$$

FPR is the rate at which malicious data are detected:

(14) $$\begin{array}{c} \text{FPR}=\frac{\left\|\text{FP}\right\|}{\left\|\text{TN}\right\|+\left\|\text{FP}\right\|}. \end{array}$$

FNR is the rate at which normal data are detected as abnormal data, which means

(15) $$\begin{array}{c} \text{FNR}=\frac{\left\|\text{FN}\right\|}{\left\|\text{TP}\right\|+\left\|\text{FN}\right\|}. \end{array}$$

#### 3.2.3. Updating the Trust Value to Detect Malicious Nodes

From the perspective of *v*_*i*_, two confidence values (weighting) are used to represent the reliability of node *v*_*i*_.

In the absence of an event, a sensor node that fails or sends a “1” signal will reduce weight, shown as follows:

(16) $$\begin{array}{c} w_{\text{ij}}^{0}=\left\{\begin{array}{cc} \max\left(0,w_{\text{ij}}^{0}-\alpha \right) & \text{for }b_{j}=1\text{ or }F_{j}=1\\ \min\left(1,w_{\text{ij}}^{0}+\beta \right) & \text{for }b_{j}=0 \end{array}\right.. \end{array}$$

Similarly, the weight of node *v*_*i*_ itself is also updated as follows:

(17) $$\begin{array}{c} w_{\text{ii}}^{0}=\left\{\begin{array}{cc} \max\left(0,w_{\text{ii}}^{0}-\alpha \right) & \text{for }b_{i}=1\\ \min\left(1,w_{\text{ii}}^{0}+\beta \right) & \text{for }b_{i}=0 \end{array}\right.. \end{array}$$

The above improvements reduce the number of sensors marked by *ɑ* as “1.” On the other hand, sensor nodes with accurate measurements will increase their pods. In this process, the node reports a “1” or maliciously reports a “1” failure, and the node with a map error of “0” gets more pods during the update process and keeps the upper limit of the override value at 1.

If an event occurs, the node in the event area should report “1,” the malicious node in the event area, and the card “0” failure node deliberately should report “0” to cause the wrong decision. The goal of the update now is to solve these problems:

(18) $$\begin{array}{c} w_{\text{ij}}^{1}=\left\{\begin{array}{cc} \max\left(0,w_{\text{ij}}^{1}-\alpha \right) & \text{for }b_{j}=0\;or\;F_{j}=1\\ \min\left(1,w_{\text{ij}}^{1}+\beta \right) & \text{for }b_{j}=1 \end{array}\right.. \end{array}$$

Similarly, the weight of node *v*_*i*_ itself is also updated as follows:

(19) $$\begin{array}{c} w_{\text{ii}}^{1}=\left\{\begin{array}{cc} \max\left(0,w_{\text{ii}}^{1}-\alpha \right) & \text{for }b_{i}=0\\ \min\left(1,w_{\text{ii}}^{1}+\beta \right) & \text{for }b_{i}=1 \end{array}\right.. \end{array}$$

When updating the weight, the two parameters *α* and *β* play an important role. They affect the detection rate and detection interval of malicious nodes. If the value of *α* is large, some nodes can be sacrificed. For a given p_t_ and p_ma_, condition P of the reported data is malicious and the probability of conflict with the actual data can be written as

(20) $$\begin{array}{c} P_{\text{inv}}=p_{t}\left(1-p_{\text{ma}}\right)+\left(1-p_{t}\right)p_{\text{ma}}. \end{array}$$

Let *N*_*d*_ be the average number of event-free periods required to detect malicious nodes. Then, for a given *α* and *β*, the response time *N*_*d*_ can be derived from the following equation:

(21) $$\begin{array}{c} N_{d}\left(P_{\text{inv }}\cdot \left(-\alpha \right)+\left(1-P_{\text{inv }}\right)\beta \right)=-1. \end{array}$$

N_d_ can be given in the following ways:

(22) $$\begin{array}{c} N_{d}=\frac{1}{P_{\text{inv}}\cdot \alpha -\left(1-P_{\text{inv}}\right)\beta }. \end{array}$$

### 3.3. Simulation Experiment Design of Sensor Data Anomaly Detection

#### 3.3.1. Distributed Detection Scheme

**Figure 3:**
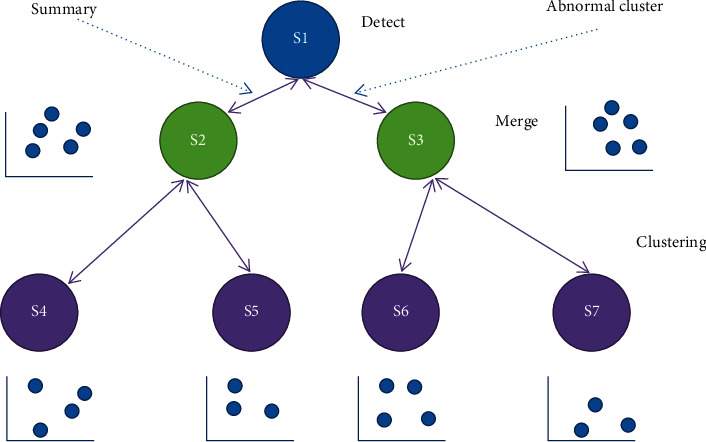
Schematic diagram of distributed anomaly detection in the WSN.

Due to the high overhead of the wireless data communication system and the heavy load of battery-powered sensor nodes, the sensors must process the data locally before sending data to reduce repetition and improve efficiency.  Figure 3 is a schematic diagram of data transmission in a distributed scheme.

Using the interconnection network, the wireless sensor network is divided into several closely interconnected networks. Each subnet operates to detect local anomalies. According to the accessibility of data transmission, efficient data combinations and data combinations are sent to the master node level as soon as possible. The master node transmits to the receiving node layer by layer. The method based on hierarchical aggregation can reduce data communication, reduce network power consumption, and effectively extend network uptime.

#### 3.3.2. Simulation Parameter Setting

The detection rate is defined as the ratio of successfully detected failure data to the total number of failures, and the false alarm rate is defined as the ratio of the number of detected good and bad data to the total number of good reads.

Table 5 summarizes the simulation parameters of two different scenarios. Scenario 1 simulates a sparse network with an average of 5 neighbors per node, whereas scenario 2 simulates an indirect distributed sensor network. The requirements of the two systems are randomly distributed. All sensor readings are generated based on the characteristics of the actual displayed data. A good sensor has a reading range of 19–20°C, whereas a defective sensor has a reading range of 25–26°C. The sensor node is randomly selected to be faulty under normal distribution, and the probability range of sensor node failure in the network is 0.05–0.23.

#### 3.3.3. Database Table Design

In order to ensure the consistency and uniqueness of the data, the data filtering team must use the deduplication function to remove the duplicate data during data collection and the same data collection device. Most data collectors are responsible for backing up and reading the original data from the sensor network. The data table structure design of data storage is shown in Table 6, including numerical data, acquisition time, acquisition equipment type, numerical equipment, temperature data, humidity data, lighting information, and voltage data. The data collection module is located at the bottom of the system hierarchy. It provides an interface to receive the initial data collected from the target sensor array and is responsible for storing and reading the initial input data. It is the basis and source of information. It is normal in the anomaly detection system.

The memResult function is responsible for storing the detection results of the anomaly detection task in the database, and the detection results can be used as a backup for future review and analysis. The data structure layout of the recording detection result table is shown in Table 7, including 5 fields: number, name, detection time, device number of the sensor under test, and abnormal detection result of the test task.

#### 3.3.4. Detection Threshold

The weighted median detection is based on the spatial correlation between the sensor data of a node and its neighbors. The working principle of the detection method is as follows.

According to the weight of the neighbor and the i-th item, calculate the average output of neighbor data:

(23) $$\begin{array}{c} {\hat{x}}_{i}=\frac{\sum_{k=1}^{j}\lambda_{\text{ik}}x_{\text{ik}}}{j}. \end{array}$$

Calculate the sensor data of the node *i*, the relative difference between xi and the median x̂_i_, namely,

(24) $$\begin{array}{c} \Delta_{2}=\frac{\left|x_{i}-{\hat{x}}_{i}\right|}{{\hat{x}}_{i}}. \end{array}$$

Decide whether to update the trust value according to the following formula:

(25) $$\begin{array}{c} \lambda_{\text{ij}}=\left\{\begin{array}{cc} \max\left(0,\lambda_{ij}-\alpha \right) & \text{if }\Delta_{2}>\theta_{2}\\ \min\left(\lambda_{\max},\lambda_{ij}+\beta \right) & \text{if }\Delta_{2}\leq \theta_{2} \end{array}\right.. \end{array}$$

The standard deviation *σ* is calculated as

(26) $$\begin{array}{c} \sigma =\sqrt{\frac{1}{T}\sum_{t=1}^{T}{\left(x_{t}-{\hat{x}}_{t}\right)}^{2}}. \end{array}$$

### 3.4. Simulation Experiment Results and Prediction Performance Analysis

#### 3.4.1. Self-Test

**Figure 4:**
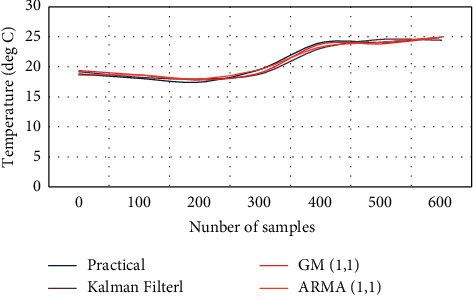
Actual data and Kalman, GM(1, 1), and ARMA(1, 1) estimated data.

This article uses the data from the real temperature sensor provided by Intel to train three time series prediction models. The real data and the values of the data estimated by the three models are shown in Figure 4.

#### 3.4.2. Results of Comparison of Detection Accuracy

In this section, the three algorithms are compared based on detection accuracy, false alarm rate, and simulation time.

**Figure 5:**
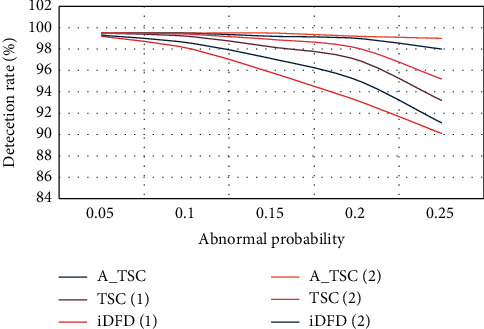
Detection accuracy of three algorithms in two scenarios.

The detection accuracy of the three algorithms in the two cases is shown in Figure 5.

It can be seen from Figure 5 that the detection accuracy of A_TSC is close to 100% in both cases, and the algorithm shows that the detection accuracy can be improved. For the other two algorithms, the accuracy of TSC and iDFD in detecting scene 2 is much higher than that of scene 1. In fact, the error detection of these two algorithms largely depends on the error of the node. Therefore, as the average number increases from 5 to 13, the detection accuracy improves. In addition, the detection accuracy of the TSC and iDFD conversion algorithm will decrease as the probability of failure increases, and the increase in the probability of failure leads to a higher ratio of faulty neighbors, which in turn will reduce the accuracy of detection. However, the downward trend of the updated A_TSC algorithm introduced in this chapter is not clear. In fact, the self-test results are input into a medium pod test program, which makes the algorithm not strictly dependent on the density of neighbors like the other two algorithms.

**Figure 6:**
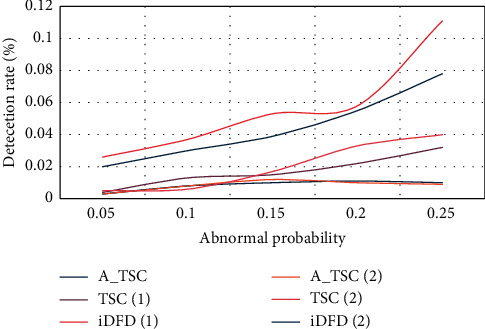
False alarm rate of three algorithms in two scenarios.

Figure 6 shows the false alarm rates of the three algorithms in scenario 1 and scenario 2.

Figure 6 shows that in both cases, the false alarm levels of the TSC and A_TSC algorithms are kept at a low level. However, as the failure probability increases and the average number of neighbors decreases, the number of iDFD false alarms also increases. The results show that, compared with the algorithm based on spatial relationship, the algorithm based on the combination of spatiotemporal correlation can reduce the false alarm rate.

## 4. Research on the Development Strategy of Rural Finance Innovation under the Internet Background

### 4.1. Problems in Rural Financial Development in the Context of the Internet

#### 4.1.1. Credit Risk

Credit risk is also called default risk, because it is in financial services where the parties to the transaction fail to fulfill the corresponding obligations stipulated in the contract due to various reasons. Farmers generally do not have a strong sense of risk, and their awareness of risk is increasing, which has become a frequent occurrence of financial fraud. Since the birth of Internet finance, many financial institutions have raised funds with high returns and illegally raised funds in rural areas. China currently does not have adequate regulatory mechanisms, which has led to frequent financial chaos. On the other hand, the risk of rural land lenders is that the agricultural industry is very unstable and vulnerable to various natural factors. Once farmers lack integrity and credit in rural areas, and the system has not yet been established, it will hinder Internet finance companies. An orderly investigation of the credit of farmers cannot guarantee that personal reputation hinders the repayment of the farmers' principal and interest, thus transferring the risk to the platform and presenting credit risk.

#### 4.1.2. Operational Risk

Operational risk is the risk of accidental losses caused by internal errors of employees, information system failures, or external failures. This risk mainly includes the following two aspects: on the one hand, the risks caused by the internal operation of the platform, because the loan officer is not familiar with the process, rural loan officers have limited education, do not have a deep understanding of financial education, and the platform is also in a period of development, lacking an effective management and supervision mechanism, which can easily cause some subjective misjudgments; on the other hand, farmers are operating themselves and China also makes mistakes. Farmers have relatively low levels of education, no knowledge of Internet finance, inability to understand business emotions, low awareness of risk prevention, insecure online payment environment, stolen passwords, and loss of property.

#### 4.1.3. Liquidity Risk

Liquidity risk refers to the risk that rural Internet financial platforms have certain debt repayment ability, but due to the lack of corresponding funds, they cannot obtain effective funds at a reasonable cost to deal with the risk of overdue debt, which affects the risk return, and the causes of liquidity risk are more diversified. It can also be classified as a global risk. Agricultural products are mainly harvested in spring and autumn, and the demand for capital and output is relatively concentrated. As a result, equity lending platform funds cannot guarantee an orderly circulation and cannot actually meet the needs of the public.

### 4.2. Countermeasures and Suggestions for Rural Financial Innovation and Development under the Internet Background

In the rapid advancement of Internet finance, a great improvement has been made, which will help promote the continuous development of the rural financial market. Rural financial institutions should be based on reality and proceed from the specific conditions of rural areas. The government should give priority to agricultural services and promote the comprehensive integrated development of Internet and intelligent financing. Specifically, it can be developed from the following two aspects: on the one hand, it is the revision of ideas and concepts, and the establishment of Internet thinking; it is clear that customers and services are the core of rural financial development, and diversified products are provided according to the financial needs of various rural entities. And services to meet their needs further improve risk prevention and awareness, and more effective judgments, further enhance user experience, improve the organizational structure and overall process, and create a development process that meets business needs. At the same time, it will absorb the development experience of other financial institutions, update its products, strengthen the use of Internet technology, and provide products that meet the needs of enterprises, demonstrate the advantages of the Internet, and better meet the needs of residents. In rural areas, rural financial institutions adopt more advanced concepts, focus on system construction, and use Internet technology to create a business processing platform for orderly processing of online and offline businesses.

In the process of continuous development of Internet technology, traditional agricultural industries have undergone great changes under the influence of Internet finance. From an objective point of view, the Internet does not exist in a formal way, but is connected with various production links. The use of Internet technology will promote the development of agriculture. Starting from the basic links of agricultural production, the P2P platform is used to meet the financing needs of farmers, and funds are obtained through land mortgages in the agricultural production links; from the perspective of land circulation, e-commerce platforms can meet the financing needs of farmers and provide targeted Support services.

Internet finance is very different from traditional finance. The latter developed earlier, has many physical outlets, and has strong financial strength. However, although Internet finance has only developed in recent years, it has developed rapidly. According to the analysis of the development status, the joint development of the Internet and traditional finance can provide complementary advantages and jointly expand the rural financial market. Therefore, rural finance must actively integrate with Internet finance, make full use of the advantages and characteristics of the Internet, develop diversified products, meet the needs of rural financial institutions, and establish a service system that adapts to rural development. The two are fully integrated to realize the exchange of information and market resources, and establish comprehensive financial service outlets that match the needs of rural customers. Promote the development of traditional financial institutions in accordance with the specific conditions of rural areas, open up more development channels, and effectively meet the individual needs of more farmers.

In the development of the rural financial market, the latest technologies such as big data and cloud sharing can be combined, and based on the target level, the rural credit research system can be established on this basis, and the development can be carried out from the following three aspects: first, through the “infrastructure,” “The construction of,” laid the foundation for the development of Internet finance; second, rural finance can provide financial services more effectively with the help of the Internet platform; third, during the development process, farmers' credit concepts will undergo certain changes and they will accept new. More diversified technologies must be built with the central bank as the core, farmers as the main body of development, and integration of new systems from different companies. In the process of building the rural credit system, the central bank must play a supervisory and management role, not only to objectively and completely understand the risks and operating conditions of financial institutions, but also to establish cooperative relations with other departments to collect an objective and complete understanding of farmers. In this way, existing resources can be integrated to play a greater role, and financial institutions can also obtain sufficient data on various platforms to cover more products. It is also very useful to improve the credit data of rural households in the credit investigation platform. Nonfinancial institutions can also master more data in the corresponding range to provide diversified services to rural households. Farmers must voluntarily participate in investigations and express their demands, which will help establish a credit system that meets the financial needs of the rural public.

## 5. Conclusion

This article will first introduce a text detection system for malicious nodes based on double standards. Each sensor point retains the reliability value of neighboring nodes to reflect the influence of previous behaviors on decision-making. In order to reduce the false alarm rate of malicious node detection and increase for the accuracy of event area detection, this program uses two levels to achieve more accurate malicious message detection efficiency without sacrificing normal nodes. The simulation results show that the two-tier program is better than the single-tier system. Next, the rural financial development background under “Internet prediction + intelligent prediction” is divided into two levels: on the one hand, it classifies and summarizes the services of traditional financial institutions such as Agricultural Bank, Postal Savings, and Rural Credit Cooperatives; on the other hand, the service functions of rural Internet financial institutions, such as the supply chain finance of agricultural leading enterprises, the entire industry chain of the e-commerce platform, rural finance, and the P2P online loan assistance platform, are analyzed.

## Figures and Tables

**Table 1:** Security requirements of wireless sensor networks.

Species	Feature	Demand details
Basic security requirements this An all	Confidentiality	Ensure that authorized users access information and prohibit unauthorized access.
	Availability	Ensure that legitimate users do not deny the abuse of data and resources.
	Completeness	When inputting and forwarding, the data are not displayed, and it is illegal to agree to modify and destroy the information to protect the consistency of the information and data.
	Authenticity	Ensure the authenticity and credibility of the identities and information of both parties communicating with each other.

Need	Data security	Unauthorized access, modification, or transmission of any data is not allowed.
	Self-healing and recovery ability	The network must have a certain degree of self-defense ability to resist unknown attacks.

**Table 2:** Security threats of sensor networks.

Field	Main threat
Data	Illegal eavesdropping, malicious data tampering, false data injection
Communication	Witch attack, black hole attack, flood attack, wormhole attack
Service	DDOS attack
Hardware	Theft, destruction, equipment hardware intrusion, natural disasters

**Table 3:** Attack characteristics of sensor networks.

Typical attack method	Attack description	Representative characteristics
Malicious data tampering	Attackers maliciously modify the collected data or reported data in the network, causing abnormalities in the data transmitted and reported by the network nodes.	Perceived data such as temperature, humidity, luminosity, and data flow information
Fake data injection	Attackers use false control data or unsupervised data to compress the sensor network, causing network failures or unreliable data reported by nodes.	Sensing data such as temperature, humidity, luminosity, and data flow information entropy

**Table 4:** Various results of anomaly detection.

Result	General data	Abnormal data
Was detected as regular	TP (regular data are received and classified correctly)	FP (abnormal data are received, classification error)
Was detected as abnormal	FN (regular data rejected, misclassification)	TN (abnormal data are rejected, the classification is correct)

**Table 5:** Simulation parameter settings.

Scenes	Scene 1	Scene 2
Network density	Discrete	Dense
Number of nodes	Twenty-four	53
Average number of neighbors	4	15
Data anomaly type	Anomaly model	Anomaly model
Abnormal probability	0.04–0.26	0.05–0.23

**Table 6:** Raw data table of the sensor network.

Serial number	Field	Type of data	Description
1	Id	Int (primary key)	The serial number, the primary key of the table, increases in sequence
2	Time	DateTime	Date and time, the format is 2020-01-01 11 : 11 : 11
3	NodeType	Int	Used to distinguish the deployment location of the data table, value 01 represents the sensor node, and value 02 represents the data control center
4	DeviceId	Int	Number of sensor device reporting data
5	Temperature	Float	Sensor collection temperature
6	Humidity	Float	Humidity collected by the sensor
7	Light	Float	Luminosity collected by the sensor
8	Voltage	Float	Device voltage of the sensor

**Table 7:** Test result table.

Serial number	Field	Type of data	Description
1	Id	Int (primary key)	The serial number, the primary key of the table, increases sequentially
2	DetectName	Varchar	The name of the anomaly detection task
3	DetectTime	DateTime	The time when the detection task was started, format
4	DeviceId	Int	For 2020-01-01 11 : 11 : 11
5	Result	Int	Device number of the detected sensor

## Data Availability

The data used to support the findings of this study are available from the corresponding author upon request.

## References

[1] Cong W., Ji L., Yuanyuan Y. (2015). Wireless rechargeable sensor networks status and future trends. *Journal of Communications*.

[2] Deng R., He S., Cheng P., Sun Y. (2017). Towards balanced energy charging and transmission collision in wireless rechargeable sensor networks. *Journal of Communications and Networks*.

[3] Zhang Y., He S., Chen J. (2017). Near optimal data gathering in rechargeable sensor networks with a mobile sink. *IEEE Transactions on Mobile Computing*.

[4] Lu W., Gong Y., Liu X., Wu J., Peng H. (2018). Collaborative energy and information transfer in green wireless sensor networks for smart cities. *IEEE Transactions on Industrial Informatics*.

[5] Yang C., Chin K.-W. (2017). On nodes placement in energy harvesting wireless sensor networks for coverage and connectivity. *IEEE Transactions on Industrial Informatics*.

[6] Han G., Jiang J., Zhang C., Duong T. Q., Guizani M., Karagiannidis G. K. (2016). A survey on mobile anchor node assisted localization in wireless sensor networks. *IEEE Communications Surveys & Tutorials*.

[7] Fantacci R., Pecorella T., Viti R., Carlini C. (2014). A network architecture solution for efficient IOT WSN backhauling: challenges and opportunities. *IEEE Wireless Communications*.

[8] Patwari N., Wilson J. (2010). RF sensor networks for device-free localization: measurements, models, and algorithms. *Proceedings of the IEEE*.

[9] Fang S.-H., Lin T.-N. (2010). Cooperative multi-radio localization in heterogeneous wireless networks. *IEEE Transactions on Wireless Communications*.

[10] Lopez T., Winkler A. (2018). The challenge of rural financial inclusion - evidence from microfinance. *Applied Economics*.

[11] Koru B., Abate G. T., Berhane G. (2019). How should rural financial cooperatives be best organized? evidence from Ethiopia. *Annals of Public and Cooperative Economics*.

[12] Sakanga V. I. R., Chastain P. S., McGlasson K. L. (2020). Building financial management capacity for community ownership of development initiatives in rural Zambia. *The International Journal of Health Planning and Management*.

[13] Grover J., Sharma S. Security issues in wireless sensor network—a review.

[14] Zheng G., Gong B., Zhang Y. (2021). Dynamic network security mechanism based on trust management in wireless sensor networks. *Wireless Communications and Mobile Computing*.

[15] She W., Liu Q., Tian Z., Chen J.-S., Wang B., Liu W. (2019). Blockchain trust model for malicious node detection in wireless sensor networks. *IEEE Access*.

[16] Zhu B., Addada V., Setia S., Jajodia S., Roy S. Efficient distributed detection of node replication attacks in sensor networks.

[17] Yang H., Zhang X., Cheng F. (2020). A novel algorithm for improving malicious node detection effect in wireless sensor networks. *Mobile Networks and Applications*.

